# Complete percutaneous coronary revascularization: An elegant solution to left ventricular dysfunction caused by severe coronary artery disease

**DOI:** 10.1002/ccr3.9224

**Published:** 2024-08-05

**Authors:** Stefan Milutinovic, Kamaldeep Singh, Stevan Oluic, Juan C. Lopez‐Mattei, Ricardo O. Escárcega

**Affiliations:** ^1^ Department of Internal Medicine Florida State University College of Medicine, Lee Health Fort Myers Florida USA; ^2^ Department of Cardiovascular Diseases Lee Health Heart Institute Fort Myers Florida USA; ^3^ Department of Internal Medicine Mayo Clinic Health System Mankato Minnesota USA; ^4^ Department of Cardiovascular Diseases Florida Heart Associates Fort Myers Florida USA

**Keywords:** chronic total occlusion, high‐risk PCI, LV dysfunction, mechanical support

## Abstract

With increased complexity in both medical comorbidities and coronary anatomy, the proportion of surgically turndown patients and high‐risk PCI will continue to rise. Impella‐assisted complex PCI can be performed with high technical success and can improve quality of life, angina score, and potentially left ventricular ejection fraction.

## Graphical abstract

Coronary angiogram pre‐ and post‐ protected percutaneous coronary intervention in a surgical turndown with a multivessel coronary artery disease.

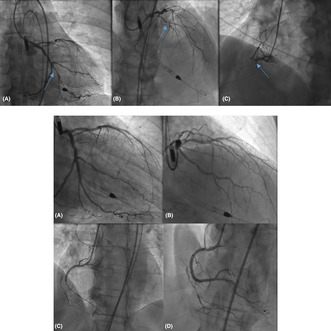

## 1 INTRODUCTION

Many patients with heart failure (HF) also have coronary artery disease (CAD), with an estimated prevalence of 50%–65%.^1^ Recent data indicate that CAD has become the most common cause of HF, surpassing hypertension and valvular heart disease.^2^ CAD significantly increases the risk of developing HF, whether it presents with reduced or preserved ejection fraction.^3^ Despite limited evidence regarding the risk of all‐cause and cardiovascular mortality, the 2021 Expert Consensus Pathway for Optimization of Heart Failure Treatment recommends coronary revascularization for appropriate patients.^4^, ^5^ Whether percutaneous coronary intervention (PCI) or coronary artery bypass surgery (CABG) is preferred, patients with complex coronary occlusions who are not suitable surgical candidates are increasingly referred for hemodynamic‐supported complex percutaneous revascularization.

## 2 CASE PRESENTATION

### 2.1 Case history

A 64‐year‐old male patient with hypertension, smoking, and alcohol use was admitted to the hospital with worsening scrotal and lower extremity swelling.

### 2.2 Differential diagnosis, investigations, and treatment

**FIGURE 1:**
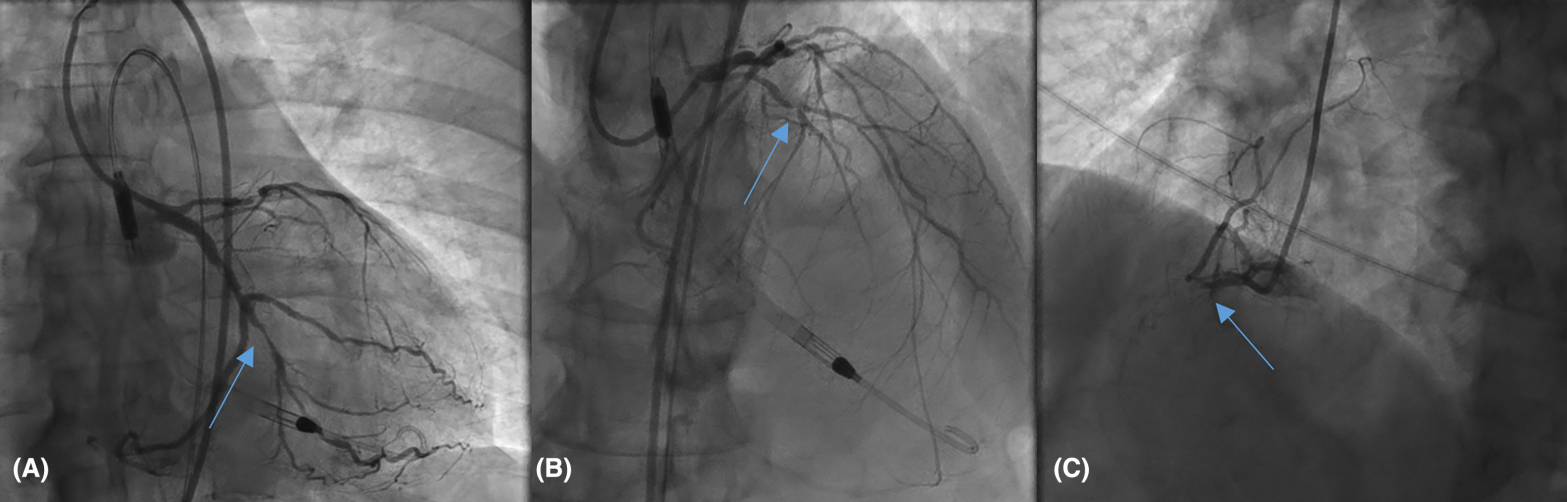
Left (A, B) and right (C) coronary systems showing multivessel coronary artery disease, including chronic total occlusion of the right coronary artery, which fills with collaterals from the left coronary system, subtotally occluded proximal calcified LAD, 70%–80% proximal and 70% mid left circumflex stenosis.

**FIGURE 2:**
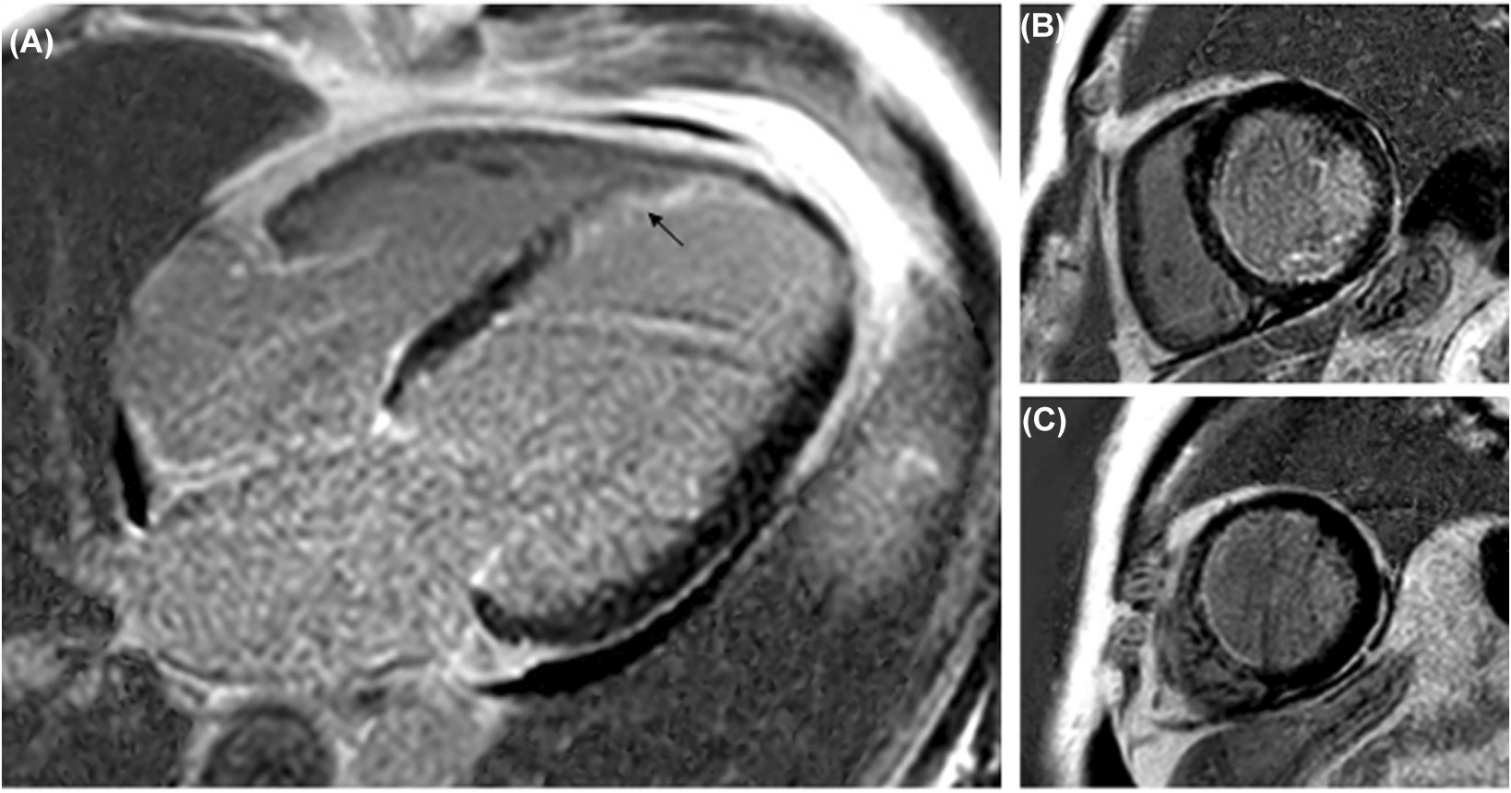
Viability assessment with cardiac MRI: Panel (A) shows late gadolinium enhancement (LGE) acquisition in four Chamber plane with a transmural infarction at distal apical septal segment (see arrow). Panel (B) shows LGE images in short axis plane with mid inferoseptal and mid inferior subendocardial infarction. Panel (C) shows proximal apical septal segments with subendocardial infarction. These findings were consistent with viable RCA and circumflex territories and predominantly viable LAD, with non‐viable distal apical septal segment.

An initial cardiac evaluation revealed elevated troponin and pro‐BNP levels. The echocardiogram showed severe global hypokinesis with a left ventricular ejection fraction (LVEF) of 20%. Coronary angiography revealed multivessel coronary artery disease, including chronic total occlusion (CTO) of the right coronary artery with collaterals from the left coronary system (Figure 1). To fully characterize the territories needing revascularization, MRI viability study was obtained, cardiac magnetic resonance (CMR) showed viable myocardium at RCA, circumflex, and predominantly viable left anterior descending coronary artery (LAD), except for distal apical septal segment. (Figure 2). Due to the severity of CAD, the patient was referred for CABG. However, given the risk for surgery in the setting of poor left ventricular function and poor target arteries for bypass surgery, he was deemed not a good candidate by the heart team.

### 2.3 Outcome and follow‐up

**FIGURE 3:**
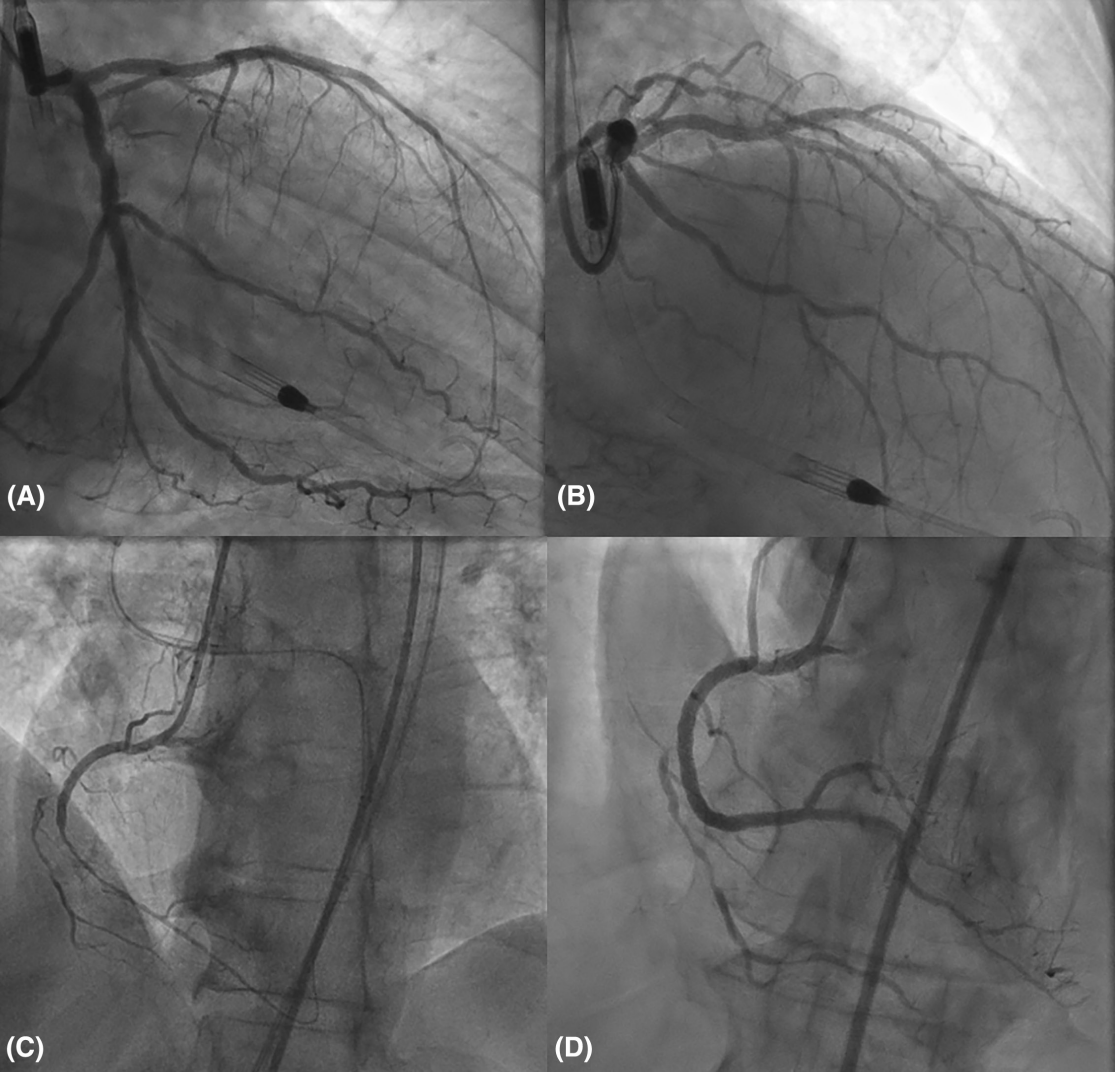
(A, B) Successful multivessel PCI with Impella support, PCI of proximal to distal circumflex with two drug‐eluting stents, and successful rotational atherectomy of proximal LAD with two drug‐eluting stents. (C, D) Successful CTO PCI in retrograde fashion of the right coronary artery.

After a thorough evaluation of his anatomy, the patient underwent percutaneous revascularization in two settings: first, multivessel PCI with Impella CP (Abiomed, Danver, Massachusetts) support (Figure 3A,B) of LAD and Circumflex. Furthermore, the patient subsequently underwent CTO PCI to achieve complete revascularization. The CTO PCI of the right coronary artery was performed via a primary retrograde approach with the placement of two drug‐eluting stents (Figure 3C,D). After complete revascularization, the patient's symptoms have improved significantly with the marked reduction in his NYHA class. Three months later, the patient was NYHA Class I and his echocardiogram showed a significant recovery in LVEF to 45%–50% (Video 1).

**VIDEO 1:** Improvement in LVEF status‐post‐multivessel PCI and CTO revascularization of RCA: Panel (A) shows a 2D Echo clip where significant segmental hypokinesis can be appreciated prior to complex coronary intervention (LVEF was 20%–25%). Panel (B) shows significant improvement in hypokinetic segments, with an LVEF of 45%–50%.

## 3 DISCUSSION

Surgical revascularization is a favorable strategy for patients with diabetes, left main disease, and multivessel coronary artery disease. However, approximately 20% of these patients may not qualify for surgery due to various reasons, such as subjective decision‐making by operators, inadequately accounted factors in surgical risk models (e.g., poor targets), exclusion from clinical trials, a historical preference for PCI as the primary treatment, and limited recommendations from societal guidelines.^4^ The main goal of the surgical approach is to ensure complete revascularization, particularly in patients receiving three or more grafts, as this has been associated with improved survival compared to those receiving fewer than three grafts.^6^ This goal has been extrapolated to percutaneous revascularization, but its applicability to stable coronary disease remains controversial due to inconsistent data. However, in patients with acute coronary syndromes and ST‐segment elevation, complete percutaneous revascularization has been shown to improve outcomes.^7^ During PCI, the completeness of revascularization is crucial, as a higher residual SYNTAX score is associated with worse 5‐year mortality.^8^

CTO is defined as the complete non‐acute occlusion of the coronary artery for at least 3 months. Coronary CTO PCI is a complex procedure that requires additional training and a deep understanding of various techniques. It involves multiple radial and/or femoral access points, adding to the complexity. There are four main strategies for crossing the CTO lesion: anterograde wire escalation, anterograde dissection and re‐entry, retrograde wire escalation, and retrograde dissection and re‐entry.^9^ Multiple wires, each with different degrees of stiffness, coating, and maneuverability, can be used to navigate through the true lumen or subintimal space. The retrograde approach involves accessing the lesion distally via collaterals and is used in more complex anatomical settings or when the anterograde approach has failed. However, it carries a higher risk of periprocedural complications. Meta‐analysis has found that PCI of CTO lesions has higher odds of in‐hospital cardiac death and cardiac tamponade compared to PCI of non‐CTO lesions.^10^ Additionally, the same meta‐analysis reported that the CTO group had a lower procedural success rate and higher major adverse cardiac outcomes during clinical follow‐up. An important limitation of CTO PCI is that procedural outcomes can be operator and center‐dependent. Higher‐volume operators (>60 CTO PCI) tend to perform this high‐complexity procedure with higher technical and procedural success rates.^11^ Nonetheless, CTO PCI is considered an essential part of complex high‐risk intervention procedures for indicated patients and is increasingly viewed as an alternative to coronary artery bypass graft surgery. Mechanical circulatory support (MCS), specifically the Impella device, has been used to facilitate complex PCI.^12^, ^13^

Hemodynamically supported revascularization is commonly performed in patients with an LVEF of ≤35%, but it is not exclusive to this group. Despite advancements in procedural techniques, equipment, and the implementation of a systematic hybrid approach, CTO PCI still faces challenges due to the high‐risk characteristics of many patients, particularly those with impaired left ventricular function.^14^, ^15^ Different technical success rates for CTO PCI have been reported in the literature. The 2021 PROGRESS‐CTO Registry demonstrates a high technical success rate of 85% and a low incidence of in‐hospital major adverse cardiac events (MACE) at 2.1%.^16^ Data have also shown that the retrograde technique, as in our case, can increase the technical success rate, but it is associated with increased rates of complications.^17^, ^18^

The effectiveness of CTO PCI in relieving angina has been well established. Following the procedure, our patient reported significant symptomatic relief at a three‐month follow‐up visit. Several studies have demonstrated improved angina symptoms following successful CTO PCI.^19^, ^20^ Compared to patients with unsuccessful CTO PCI, the OPEN‐CTO registry reported statistically significant improvements in the Seattle Angina Questionnaire Quality of Life Score and Rose Dyspnea Scale score.^21^ Similarly, the European CTO Club reported improvements in dyspnea and angina after a follow‐up period of 23 months.^22^

Although data has significant limitations, CTO PCI does not appear to affect all‐cause mortality (one‐year relative risk [RR] 1.70, 95% CI 0.50–5.80, and four‐year RR 1.77, 95% CI 0.19–16.06), myocardial infarction (one‐year RR 1.01, 95% CI 0.43–2.36, and four‐year RR 1.46, 95% CI 0.75–2.87), or cardiovascular disease mortality (one‐year RR 1.14, 95% CI 0.38–3.40, and four‐year RR 2.05, 95% CI 0.8–5.28) when compared to optimal medical management.^22^ This finding aligns with the limited benefits of PCI revascularization in stable coronary artery disease.^23^ It is important to note that this population, not eligible for surgery, has a high comorbidity burden that impacts mortality. Another consideration is that revascularization with hemodynamic support may be associated with a higher occurrence of in‐hospital major adverse cardiac and cerebrovascular events (MACCE), which is likely due to the selective use of mechanical support in patients with higher risk profiles.^24^

Data regarding LV systolic function is also inconsistent. The recent REVIVED‐BCIS2 trial failed to show benefits in all‐cause mortality (HR 0.99, 95% CI 0.78–1.27) or LV systolic function at 6 months (mean difference, −1.6 percentage points; 95% CI, −3.7 to 0.5) and at 12 months (mean difference, 0.9 percentage points; 95% CI, −1.7 to 3.4) compared with optimal guideline‐directed medical therapy, regardless of baseline viability characteristics.^25^ Notably, about 30% of modalities used to assess viability were other than CMR. In contrast, the RESTORE‐EF observational study reported significant improvement in LVEF at 90 days, from 35 ± 15% to 45 ± 14% (*p* < 0.0001).^26^ Additionally, this study revealed a substantial relative reduction of 76% in NYHA class III/IV heart failure symptoms and an impressive relative reduction of 97% in CCS angina class III/IV symptoms from baseline to the post‐PCI period. The improvement in LVEF was observed exclusively in patients with LVEF below 45%, whereas the improvement in heart failure and anginal symptoms was noted across the entire spectrum of LVEF. The disparity between these two trials is likely due to differences in the study populations. In the later trial, patients had higher rates of left main disease and more extensive coronary disease. Angina was also more frequent and severe. Additionally, in the REVIVED‐BCIS2 trial, two‐thirds of the patients had not been hospitalized in the previous 2 years, and there were no cases of non‐ST elevation myocardial infarction.

In conclusion, our case highlights the potential benefits of complex high‐risk PCI with mechanical support and the potential benefits of CTO PCI to improve LV function.

## AUTHOR CONTRIBUTIONS

**Stefan Milutinovic:** Conceptualization; resources; writing – original draft. **Kamaldeep Singh:** Resources; supervision; writing – review and editing. **Stevan Oluic:** Conceptualization; resources; writing – original draft. **Juan C. Lopez‐Mattei:** Supervision; visualization; writing – review and editing. **Ricardo O. Escárcega:** Supervision; writing – review and editing.

## FUNDING INFORMATION

None.

## CONFLICT OF INTEREST STATEMENT

The authors report no relationships that could be construed as a conflict of interest.

## CONSENT

Written informed consent was obtained from the patient to publish this report in accordance with the journal's patient consent policy.

## Data Availability

The data that support the findings of this study are available on request from the corresponding author. The data are not publicly available due to privacy or ethical restrictions.
